# Modular soft pneumatic actuator mimics elephant trunk locomotion

**DOI:** 10.1038/s41598-024-74105-0

**Published:** 2024-10-15

**Authors:** Ahmad R. Elchrif, Mohammed I. Awad, Shady A. Maged, Amna Ramzy

**Affiliations:** 1 Mechatronics Engineering Department, Faculty of Engineering, Ain Shams University, Cairo, 11517 Egypt; 2 Material Engineering Department, Faculty of Engineering and Materials Science, German University in Cairo, Cairo, 11835 Egypt

**Keywords:** Biomedical engineering, Mechanical engineering

## Abstract

Robots support and facilitate tasks in all life fields. Soft robots specifically have the advantages of inherent compliance, safe interaction and flexible deformability. Soft pneumatic network (Pneu-Net) is a soft pneumatic actuator (SPA) composed of network of chambers that is actuated by pneumatic power. Soft Pneu-Net fits the human interface applications perfectly. In this paper, a bio-inspired modular based design for Pneu-Net actuator is developed. The actuator mimics the elephant trunk curling to be employed for rehabilitation of human hand fingers. The actuator is an integrated four Pneu-Net modules actuator which is attached to hand’s finger. The main introduced advantages in the new developed actuator are: providing four degrees of freedom (DoF) essential for finger’s motion by single compound actuator and developing a methodology for a modular soft Pneu-Net actuator that is efficiently reproducible. The actuator’s design is developed using computer aided design (CAD) software SOLIDWORKS. The design is simulated using finite element modeling (FEM) software ABAQUS. Fabrication process uses 3D printed molds. Soft material is molded in the 3D printed molds, forming actuator’s modules. Actuator’s modules are integrated by adhesion using the soft material. A proposed non-standard hyper-elastic material biaxial tension test is introduced as a quick material properties identification method that can produce a test table used for material identification in the FEM. Enhanced version for the actuator uses reinforcement fibers. Results show advances for the reinforced actuator, as it limits the unwanted actuator’s strain and deformation. The reinforced actuator shows improved energy efficiency reaches to 46%.

## Introduction

In soft robots, rigid links are replaced by compliant materials which provide safe human interfacing and good deformability. These advantages permit soft robots a pioneer position in many applications. Human rehabilitation and exoskeleton applications have great advances, thanks to soft robots and soft actuators. Soft actuators can be classified according to the type of stimulation. Wide variety of soft actuators are developed using pneumatic networks which use pneumatic power to actuate network of cavities designed to generate directed motion^1,2^, it is also called soft fluidic actuators (SFA) or soft pneumatic actuators (SPA), fiber reinforcement or fabric reinforcement techniques are used in some designs to provide enhanced performance for the pneumatic actuators^3,4^ or even other techniques like 3D printing^5^. Other driving power or stimuli of the actuator can be electrical energy, magnetic, thermal, photo-response, or even explosives in some actuator designs^6,7^. Pneumatic networks or shortly Pneu-Nets are soft actuators composed of networks of chambers. The actuator is strained when pressure is applied causing directed motion. This motion can be controlled through the network chamber’s geometry, material properties and the amount of applied pressure. The geometry of the actuator’s chambers affects the motion direction and the range of motion for the actuator. The actuator’s network cross section and longitudinal section shapes can increase and decrease the range of bending^1^. Even the geometry shape specifies the motion characteristic whether bending, twisting or elongation^2^. Reinforced Pneu-Net enhances the demanded motion and limits the unwanted strain, which can be done with fibers or plastic like 3D printed parts^1^.

Many of the soft robotic locomotion essentially mimic the biological creatures’ behavior. Biological patterns of motion created in elephant trunk, octopus tentacles, chameleon tail and caterpillar crawling are inspirable for soft robotic actuation systems^8–10^. This biomimetic inspiration gives safe interactive advantage for the soft robotic actuators for employment in human interface applications. One fit application for the soft actuators is the rehabilitation of human hand and fingers. In some injuries, neurological conditions or as consequences of stroke, subject loses hand functionality and control. Rehabilitation is performed by physical exercising that enhances brain response and neuroplasticity responsible for organ’s muscle interaction, and accordingly organ’s motor-ability and sensory functions are improved. Soft hand gloves are designed to be used for physical therapy, which increases the availability of the tool that can be used to do exercises. Accordingly, independent and more frequent exercising sessions can be performed by the subject. Additionally, these gloves can be used as assistive tool not only exercising tool^3,11–17^.

Human finger moves in four directions; flexion, extension, adduction and abduction. Accordingly, four motions per finger are required for a fully functioning assistive tool. In this paper, an elephant trunk inspired modular based design is adopted to develop four degrees of freedom (DoF) soft Pneu-Net actuator, which supports finger rehabilitation. The modular design will facilitate the customized reproduction of the actuator, since customization is required due to the range of differences between subjects’ finger sizes and required power for assistance; moreover, for a single subject’s hand tailored actuators will be required to fulfill the differences for every finger’s size and required actuation power. The following presentation shows the order of procedures that can be followed to develop customized soft Pneu-Net efficiently. Actuator design passes by visualization stage through 3D modeling using CAD software (SOLIDWORKS). The 3D model is imported for motion simulation to 3D simulation and FEM software (ABAQUS). Modules are fabricated utilizing modular 3D printed molds. The molded actuator modules are finished by removing appendages and actuator’s modules are integrated by adhesion. The results of the FEM and experimental testing are discussed and a proposed non-standard biaxial tension test for material identification is introduced. Moreover, reinforcement fibers are introduced during the modules integration for performance improvement. Finally, the conclusion and the future work recommendations are highlighted.

## Methods

### Modular soft pneumatic networks actuator

Developing a hand wearable tool for fingers’ rehabilitation, an employed actuator shall provide the finger’s bending motions; flexion, extension, adduction and abduction. Elephant’s trunk is a pure muscle organ that has over 40,000 individual muscles; by curling, the trunk as a limb attains its bending motions. Being inspired by elephant’s trunk locomotion, a modular soft Pneu-Net actuator design is adopted. The actuator design is characterized by two main properties; being soft analogizing the pure muscles elephant’s trunk attaining four bending motions and modular. The modular design will result in enhancing the actuator’s reproduction procedures and accordingly, the execution time. Since there are many varying parameters from person to another, even the single hand has varying fingers’ sizes; so, the modular design aims to minimize the required changes that are performed only to a single module, this module will be tailored then instantiated forming the compound actuator. The actuator’s development procedures are defined by four successive steps; 3D modeling of the design, actuator’s model simulation and analysis, actuator fabrication and actuator testing; as outlined in the following subsections.

### 3D modeling

The 3D modeling for the modular actuator is based on single unit that is multiple instantiated and assembled forming the overall actuator. Accordingly, the development and reproducibility of the actuator are simplified; this can be illustrated in two aspects; the actuator’s 3D modeling and the fabrication mold 3D model. The actuator’s 3D model is created using SOLIDWORKS depending on single part which is the “module” that can be easily modified and reproduced satisfying required figure’s parameters, the module is then used to assembly four instances forming the final actuator’s 3D model. Additionally, the fabrication mold’s 3D model is required for the module only; this mold can be used to produce four copies of the module successively which can be adhered together forming the complete modular actuator.

#### Actuator 3D modeling

**Fig. 1:**
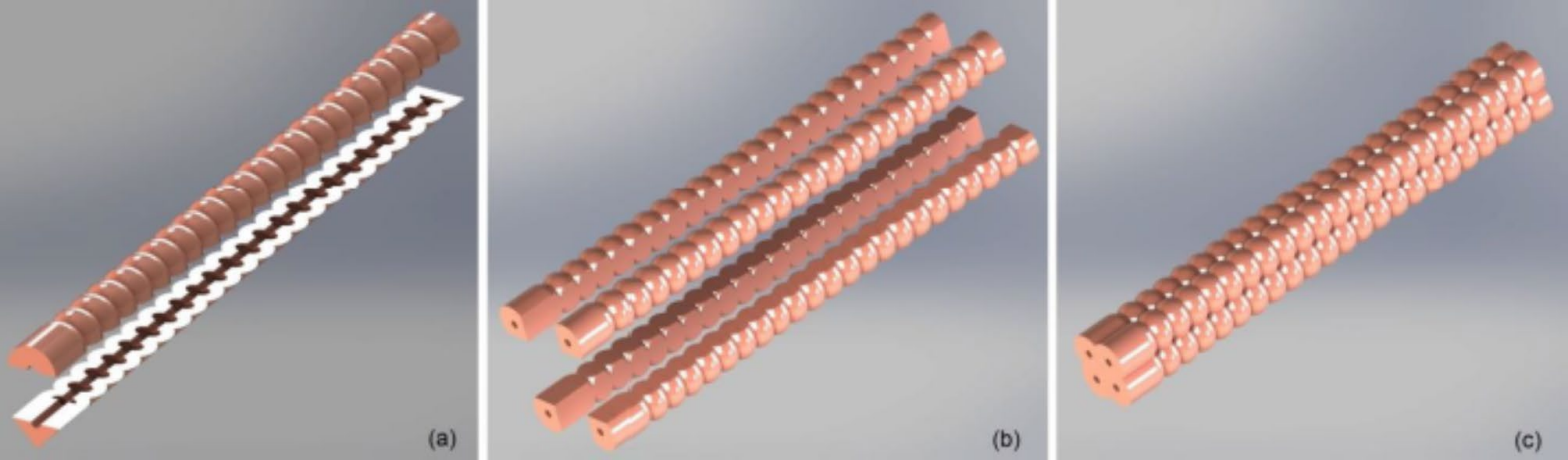
(**a**) Actuator module is sectioned showing the internal pneumatic network. (**b**) Four similar modules are shown exploded separately. (**c**) Compound actuator formed from the modular actuator to produce the four acting Pneu-Net actuator.

**Fig. 2:**
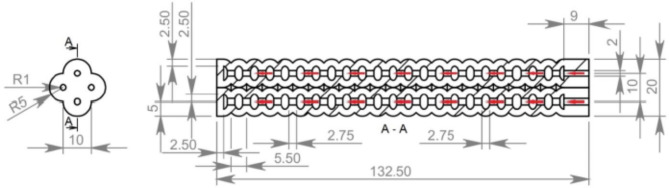
Actuator’s dimensional views show internal pneumatic networks (all dimensions are in mm).

Simplifying the analysis of the actuator’s design to the module as a soft pneumatic actuated network, as it was validated in literate research work that the actuator’s chamber cross section and the longitudinal section shape have significant influence in the actuator’s response. Considering the circular section shapes have better bending with less pressure^1^. The actuator’s module is modeled in two parts attached later; the actuator’s module is composed of 22 circular longitudinal and cross-sectional chambers. With each module has its separate air inlet, four identical modules are assembled together forming the compound actuator’s 3D model as illustrated in Fig. 1. The over-all dimensions of the actuator are 132.5 mm length, 17 mm width and height to be convenient for attachment over human’s index finger; detailed dimensional drawing for the actuator is illustrated in Fig. 2. Providing four DoF in a compound actuator is considered a new introduction in this design, since it is found in the literature that most of the previous work is concerned by introducing a single bending DoF or at most two DoF in a single actuator fulfilling the finger’s flexion and extension. However, concerned works to achieve adduction and abduction used another separate actuator.

#### Mold 3D modeling

**Fig. 3:**
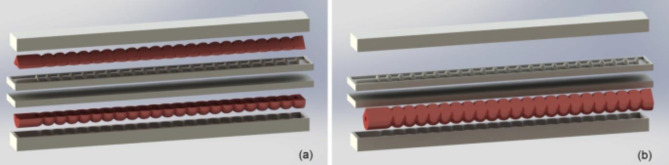
(**a**) Mold consists of four white colored parts and the actuator’s module is molded in two separate parts in pink color. (**b**) Actuator’s module parts are adhered together after molding.

Mold 3D modeling is performed to simulate the fabrication process. Having a single copy of mold that can develop the actuator module is sufficient, thanks to the modular based actuator’s design. Mold can be used to instantiate the required number of similar actuator’s modules that can be adhered together forming the multi degrees of freedom modular actuator. Module is developed in two steps, firstly two parts are molded as illustrated in Fig. 3a, and then the two parts are adhered together forming the actuator’s module, same as shown in Fig. 3b. By repeating the same process of module fabrication, four identical modules are developed and by adhering them together the compound actuator is finally produced.

### Actuator simulation

The actuator’s bending motion is simulated and analyzed by FEM using ABAQUS software. At the beginning, the actuator’s 3D model produced using SOLIDWORKS is imported to ABAQUS as a STEP file, then parts are assembled and the surfaces are identified; surfaces’ identification importance appears during applying the loads affecting the model’s simulation. Later on, silicon rubber material properties are defined. Hyper-elastic material properties for silicon rubber are defined adopting different models^18^; the model parameters can be determined manually if available. As an alternative, material testing tables can be imported to ABAQUS after defining the proper hyper-elastic model; accordingly, the software can define the model parameters. Two hyper-elastic models are used, the first one used to define Platsil Gel-A 10 silicon rubber is Yeoh second order model.

$$\:W={C}_{10}\left({I}_{1}-3\right)+{{C}_{20}({I}_{1}-3)}^{2}$$

where C_10_ and C_20_ are material constants (0.08202 MPa and 0.0000969 MPa, respectively) I_1_ is the first strain invariant.

**Fig. 4:**
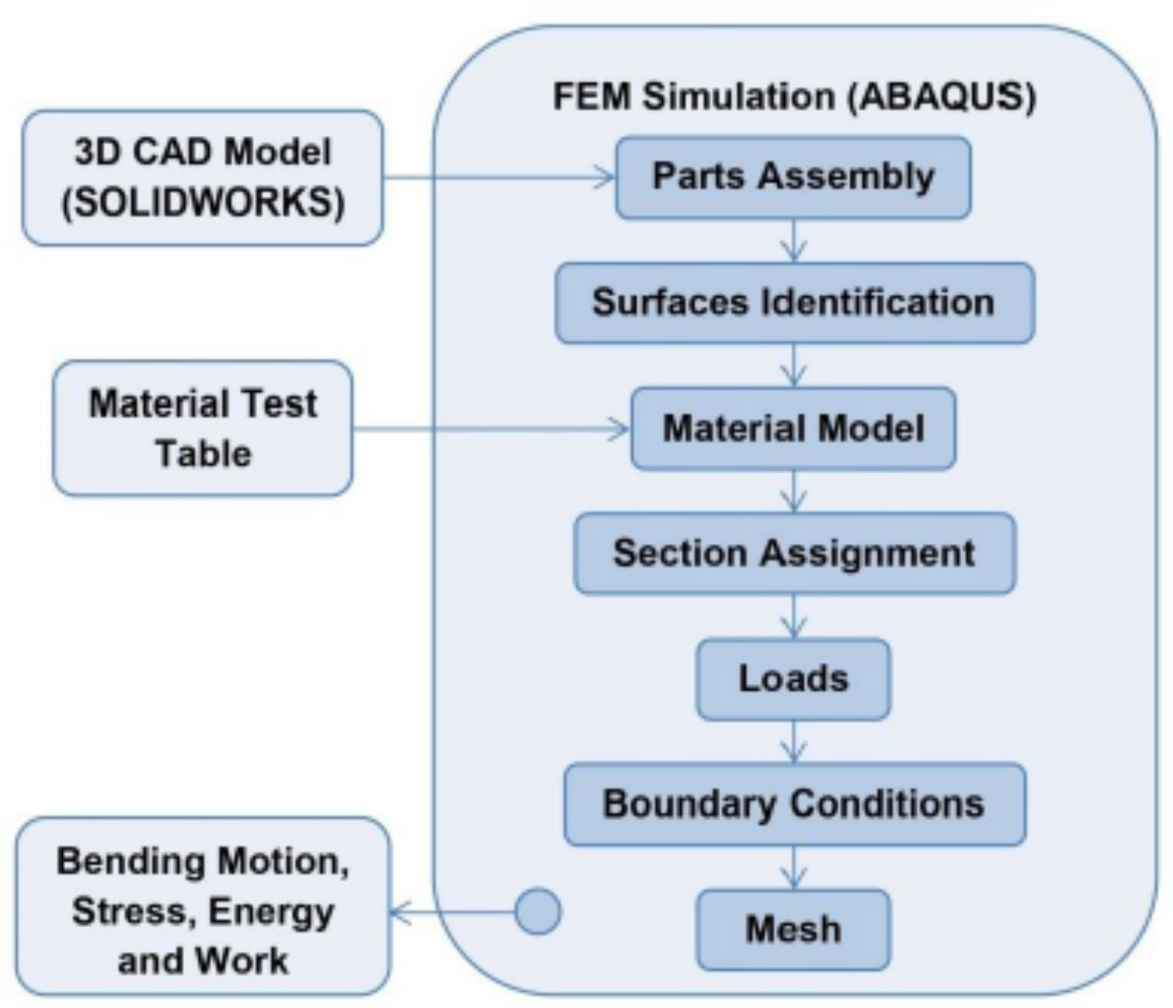
Summarized procedural flow for FEM.

In addition to defining the density as 1.1 kg/${\text{c}\text{m}}^{3}$. However, the second hyper-elastic model used is Mooney-Rivlin, will be detailed in “Actuator molding” section. After material identification, the 3D model sections are assigned to the defined materials. In the following step, the load steps and interactions are defined. Where, two steps are set; first one includes gravity load in addition to defining the boundary condition to define fixed layer on the networks air inlets, the boundary condition is defined as ENCASTRE. In the second step defined the pressure load acting on the internal cavity of the selected actuator. The external walls interactions are identified as well for outer walls contacts. Each of the four networks can be actuated alone or in combination. Actuations of each two adjacent actuators are used for bending to attain the four motions of finger: flexion, extension, adduction and abduction. The last steps for the FEM are the actuator’s mesh; the mesh element is Tet (refers to tetrahedral) with approximate global size of 2, and finally the job creation and running the simulation to get the analysis results. Figure  4 summarizes the flow of procedures for the FEM simulation process.

## Simulation results

**Fig. 5:**
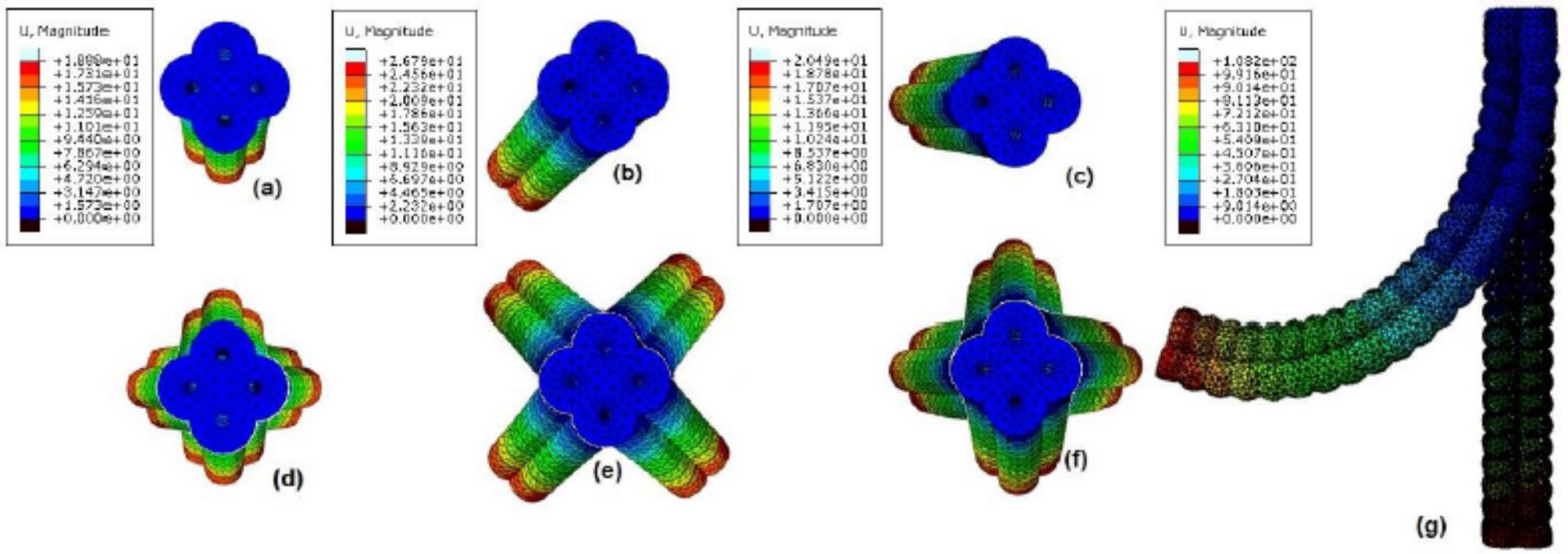
(**a**) Single network actuated by 100 kPa showing little bending. (**b**) Dual adjacent networks equally actuated by 100 kPa. (**c**) Triple networks are equally actuated by 100 kPa. (**d**) Combined shot for actuating every single network by 100 kPa. (**e**) Combined shot for actuating every dual adjacent networks equally by 100 kPa. (**f**) Combined shot for actuating every triple networks equally by 100 kPa. (**g**) Side view shot for the equally actuated dual adjacent networks by 127 kPa.

FEM analysis shows the elephant’s trunk inspired bending motion profile for the modular soft actuator illustrated in Fig. 5. In Fig. 5a, a single network is actuated. However, dual adjacent networks are equally actuated in Fig. 5b. Figure 5c shows the equal pressure actuation for triple networks. Figure 5d illustrates a combined shot for 4 alternative results when a single network is actuated; accordingly, 4 different bending direction can be resulted. Similarly, Fig. 5e illustrates a combined shot for 4 alternative results when dual networks are equally actuated. Furthermore, Fig. 5f illustrates a combined shot for 4 alternative results when triple networks are equally actuated. At this point it is good to highlight that, more alternative bending can be achieved by controlling the pressure inputs to every actuator’s network separately, and now obviously the actuator’s locomotion profile is resampling that of elephant’s trunk. However, Fig. 5g shows dual adjacent networks equally actuated. After the overview illustrated by Fig. 5, the compound actuator’s preliminary motion simulation shows the four bending DoF which are introduced by the design as a new input, however examples in the reviewed work introduced single or at most dual bending DoF^1,19^.

**Fig. 6:**
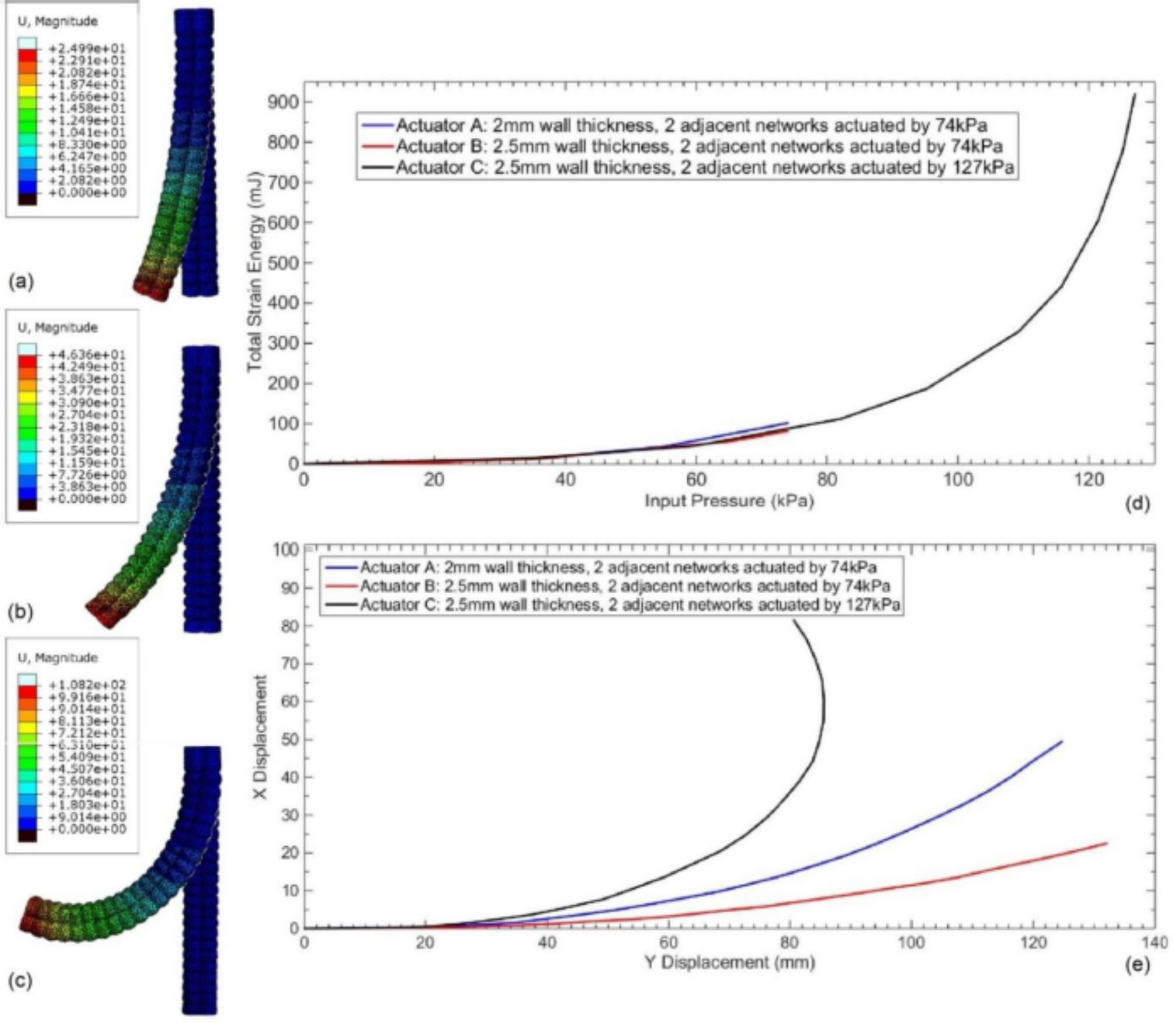
(**a**) 2.5 mm thickness actuator simulation runs for dual network actuated at 74 kPa and corresponding magnitude. (**b**) 2 mm thickness actuator simulation runs for dual network actuated at 74 kPa and corresponding magnitude. (**c**) 2.5 mm thickness actuator simulation runs for dual network actuated at 127 kPa and corresponding magnitude. (**d**) Total strain energy for the 3 simulation runs. (**e**) X–Y axes displacement for bending motion of the actuators in the 3 simulation runs.

As a preliminary consideration, actuation pressure of 100 kPa is foreseen as the safe limit for actuator-human interaction; accordingly, to select suitable actuator’s wall thickness, two samples of actuator are modeled one has wall thickness of 2 mm and the other one has 2.5 mm, while fixing all other design parameters. Preliminary observation is that; the actuator with 2 mm wall thickness simulation run is completed only up to load of 74 kPa pressure. However, the actuator with 2.5 mm wall thickness simulation run is completed up to load of 127 kPa pressure, exceeding the required limit 100 kPa with a reasonable design margin; as thickness increase allows the actuator to withstand more pressure. However, mutual comparison of the performance of the two actuators is simulated at equally applied load of 74 kPa pressure; the simulation run shows that the actuator with 2 mm wall thickness bends more than the actuator with 2.5 mm wall thickness; due to lower resistance corresponding to lower wall thickness. The results comparison is illustrated in Fig. 6; where the bending magnitude represented by (U in mm) for the 2.5 mm wall thickness actuator at 74 kPa pressure in Fig. 6a is lower than the corresponding bending magnitude for the 2 mm wall thickness actuator in Fig. 6b. Additionally, the total strain energy computed during the load application for the 2 mm wall thickness actuator is more than the corresponding total strain energy for the 2.5 mm wall thickness actuator; this higher strain energy for the 2 mm wall thickness actuator proofs its lower resistance for the bending. On the other hand, Fig. 6c shows the results for the bending magnitude represented by (U in mm) for the 2.5 mm thickness actuator at 127 kPa, which cannot be simulated for the 2 mm thickness actuator; the attained bending magnitude and total strain energy corresponding to the 2.5 mm thickness actuator at 127 kPa is much higher. Figure 6d shows the total strain against the input pressure for the three simulation runs, and Fig. 6e clarifies the bending motion as values in X and Y axes for the three simulation runs at the full loads. As a conclusion, the 2.5 mm wall thickness actuator is selected to withstand higher air pressure that can reach 100 kPa in normal operation, which is the maximum operation limit for the actuator, which is considered as a safe value with human interaction and considering the margin of 27 kPa above the 100 kPa as design safety factor.

### Actuator fabrication

The modular Pneu-Net soft actuator design cares about manufacturability and reproducibility. Each of the four modules of networks is fabricated separately then the four modules are collected and adhered together forming the final compound actuator. 3D printed mold is used to mold the silicon rubber material used for module fabrication.

#### Mold fabrication process

**Fig. 7:**
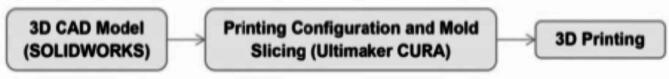
Mold development procedures.

The mold is also designed as a modular mold for fabrication simplicity and manufacturability. Firstly, the 3D modeled mold created through SOLIDWORKS CAD software is exported as stereography file (STL file). Secondly, slicing software (Ultimaker CURA 4.5.0) is used to configure the printing parameters and slice the 3D model, where PLA material is used with 100% in fill and printing temperature of 200 °C. Finally, G-Code is conducted through the (Ultimaker CURA 4.5.0) software and ready for direct printing using 3D printer. Figure 7 shows the mold printing work flow.

#### Actuator molding

Silicon rubber; locally available with shore hardness A10 is used in place of Platsil Gel-A 10 silicon rubber that used for simulation, which is mixed for around 2–3 min. with hardener compound in the ratio of 100:4 by volume. Operation time for the hardener is about 30 min. The silicon rubber is injected to the mold using syringe or just poured in. Air bubbles are removed continuously from the molded volume during the first 30 min. either by using vacuum chamber or vibrator or knocking the mold sides. After 6–8 h the part is cured then touch up for minor defects like appendages or porosity is carried out to have completed actuator network module. Four similar modules are then adhered together using the same silicon rubber material forming the compound actuator. Preliminary testing of the fabricated 2.5 mm wall thickness actuator and comparing the actuator’s bending to the simulation results at the same pressure load of 74 kPa; there was big deviation in results which is expected to be mainly due to the difference in material properties. So as a way to have a quick identification methodology for the available material, a non-standard test is proposed.

**Fig. 8:**
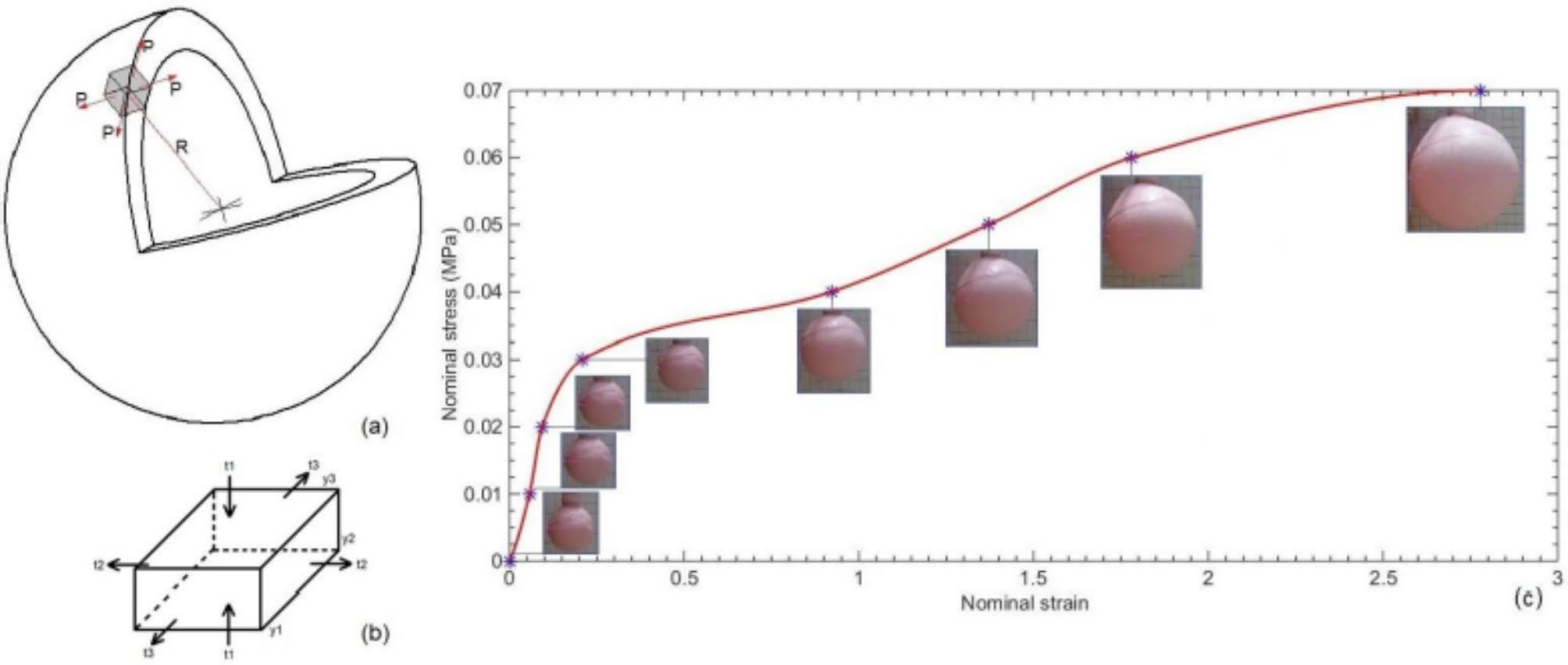
(**a**) Non-standard biaxial tension test hollow sphere. (**b**) Stresses acting on a finite section of the hollow sphere. (**c**) Experimental testing stress strain curve from the proposed non-standard biaxial tension testing.

Biaxial tension test is one of the tests that are performed to define the material properties for hyper-elastic materials, where square shaped specimen is equally tensioned in the two planer axis. Biaxial tension test data can be introduced to ABAQUS as material identification methodology. As a quick testing proposal for a non-standard biaxial tension test, assuming that for a small area on a spherical shell, the area can be simplified as a planer area illustrated in Fig. 8a. By blowing the sphere, equal pressure stretches the sphere’s wall. The assumed finite planer area on the sphere’s wall experiences equal orthogonal tension stresses equivalent to the pressure of blowing and tangent to the sphere’s wall. Describing the equivalent uniaxial compression for equi-biaxial extension (inflation); it is illustrated that experimental values for force in the plane of the sheet may be used to calculate the equivalent compressive stress in the corresponding uniaxial compression^19^. Referring to Fig. 8b for equi-biaxial extension (inflation), rubber is stretched by equal amounts of strain in two perpendicular directions (y2 and y3) and considering constant volume so,

$${{\text{y}}_{\text{1}}}*{{\text{y}}_{\text{2}}}*{{\text{y}}_{\text{3}}}\,=\,{\text{1}},$$

$${{\text{y}}_{\text{2}}}\,=\,{{\text{y}}_{\text{3}}}={\text{ }}{\left( {{{\text{y}}_{\text{1}}}} \right)^{ - 0.{\text{5}}}}$$

Considering the equation represents the true stress is the force per unit strain area,

$${\text{t}}\,=\,{\text{f}}/{\text{A}}$$

So, to calculate the force (f) acting on a section of unit length in the strained state for thin sheet (like inflated balloon) cut at right angles to the plane of the sheet and if the original thickness is (d), so area on which (f) acts on is;

$${\text{A}}\,=\,{{\text{y}}_{\text{1}}}*{\text{d}},$$

$${\text{f}}\,=\,{{\text{t}}_{\text{2}}}*{{\text{y}}_{\text{1}}}*{\text{d}}\,=\,{{\text{t}}_{\text{2}}}*{\left( {{{\text{y}}_{\text{2}}}} \right)^{ - {\text{2}}}}*{\text{d}},$$

$${{\text{t}}_{\text{2}}}\,=\,{\text{f}}*{\left( {{{\text{y}}_{\text{2}}}} \right)^{\text{2}}}/{\text{d}}$$

Superposition of a hydrostatic pressure—true stress in the plane of inflated sheet (t_2_) reduces the tensile stresses (t_2_ and t_3_) to zero and gives a resultant compressive stress (t_1_) normal to the surface of the sheet of the same magnitude; so,

$${{\text{t}}_{\text{1}}}\,=\,{\text{f}}*{\left( {{{\text{y}}_{\text{2}}}} \right)^{\text{2}}}/{\text{d}}\,=\,{{\text{t}}_{\text{2}}}$$

Since, t_1_ is the compressive stress which is equivalent to the applied pneumatic pressure (P); so,

$${{\text{t}}_{\text{1}}}\,=\,{{\text{t}}_{\text{2}}}\,=\,{{\text{t}}_{\text{3}}}={\text{ P}}$$

The proposed test uses the silicon rubber hollow sphere to be gradually blown, and Mooney–Rivlin hyper-elastic model is adopted in ABAQUS for material properties identification, assuming Poission’s ration of 0.5. The nominal stress is represented by the applied pressure steps and the nominal strain is the corresponding mean radius strain, since the strain is represented by the change in the sphere circumference.

$$\:\frac{{\Delta\:}\text{C}\text{i}\text{r}\text{c}\text{u}\text{m}\text{f}\text{e}\text{r}\text{e}\text{n}\text{c}\text{e}}{\text{i}\text{n}\text{i}\text{t}\text{i}\text{a}\text{l}\:\text{c}\text{i}\text{r}\text{c}\text{u}\text{m}\text{f}\text{e}\text{r}\text{e}\text{n}\text{c}\text{e}}=\:\frac{{\Delta\:}\text{m}\text{e}\text{a}\text{n}\:\text{r}\text{a}\text{d}\text{i}\text{u}\text{s}}{\text{i}\text{n}\text{i}\text{t}\text{i}\text{a}\text{l}\:\text{m}\text{e}\text{a}\text{n}\:\text{r}\text{a}\text{d}\text{i}\text{u}\text{s}}$$

The sphere radius is measured from photos corresponding to pressure recorded using pressure sensor using GetData Graph Digitizer application. The output stress strain curve is shown in Fig. 8c.

## Experimental results

**Fig. 9:**
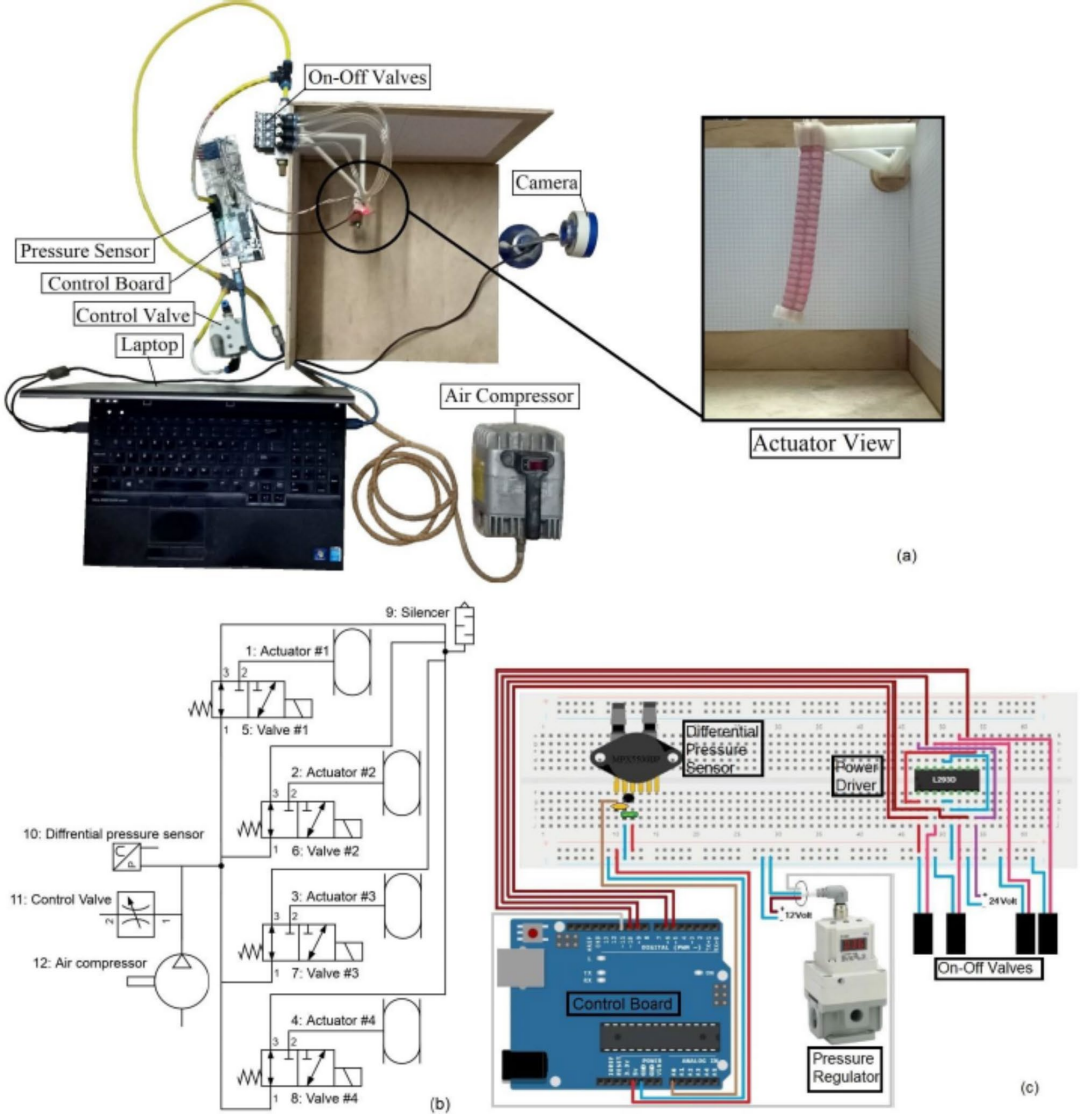
(**a**) Test arrangement. (**b**) Pneumatic control circuit. (**c**) Control circuit.

**Fig. 10:**
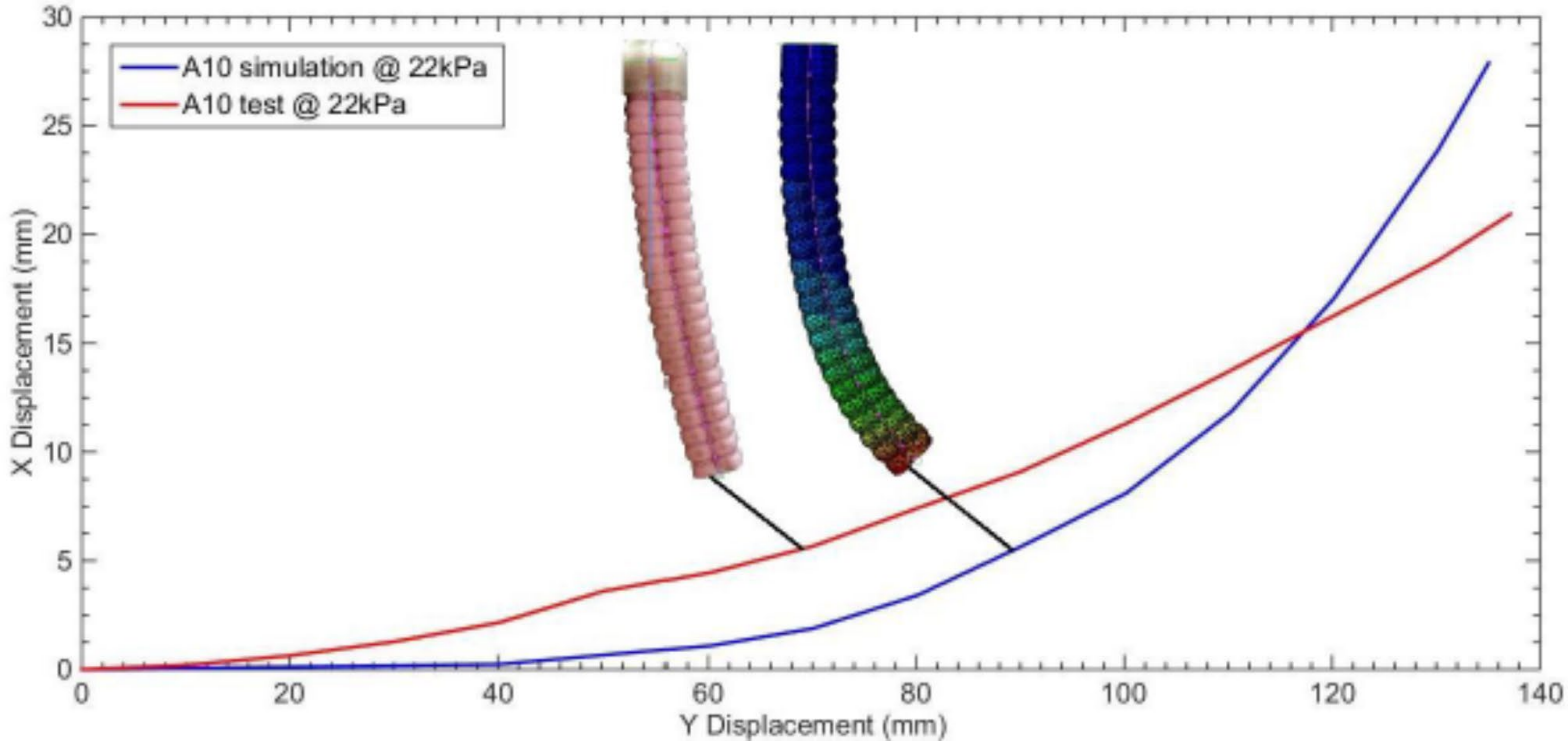
X–Y axes displacement for the bending curves of the actuator’s test model and simulation model where dual adjacent networks are actuated by 22 kPa.

Testing the actuator is performed by injecting pneumatic power to the actuator’s ports. Figure 9a shows the testing arrangement, where an Arduino Uno board is used for control prototyping which is connected with laptop, in addition to a camera which is used for capturing the vertically hanged actuator bending motion. In order to control the required motion a pneumatic circuit is used as illustrated in Fig. 9b; where, a small sized air compressor is used to produce pressurized air, a pressure sensor is used to record pressure measurements, a pressure control valve with proportional control is used to control the pressure limit in the circuit and on-off valves are controlled by pulse width modulation (PWM) output from Arduino board to introduce the pressurized air into the target networks. Figure 9c shows the control circuit for the Arduino board and connections with the differential pressure sensor and valves. A new simulation run that incorporated the non-standard proposed material test data is compared with the fabricated model with pressure equals 22 kPa introduced to dual adjacent networks as illustrated by the X–Y displacement curves shown in Fig. 10, where the simulated and tested actuator models’ images are analyzed using GetData Graph Digitizer application and final curves are represented.

The results displayed in Fig. 10 are converged in comparison with the preliminary tests’ curves that used blindly similar material parameters; since the X-axis displacement deviation is 33.3% compared to 82.2%, the Y-axis displacement deviation is 1.9% compared to 8.6% and the resultant magnitude deviation is 1.85% compared to 15.87%. Accordingly, the non-standard biaxial tension test is considered a quick approach for material identification with a compromised accuracy of simulation result.

**Fig. 11:**
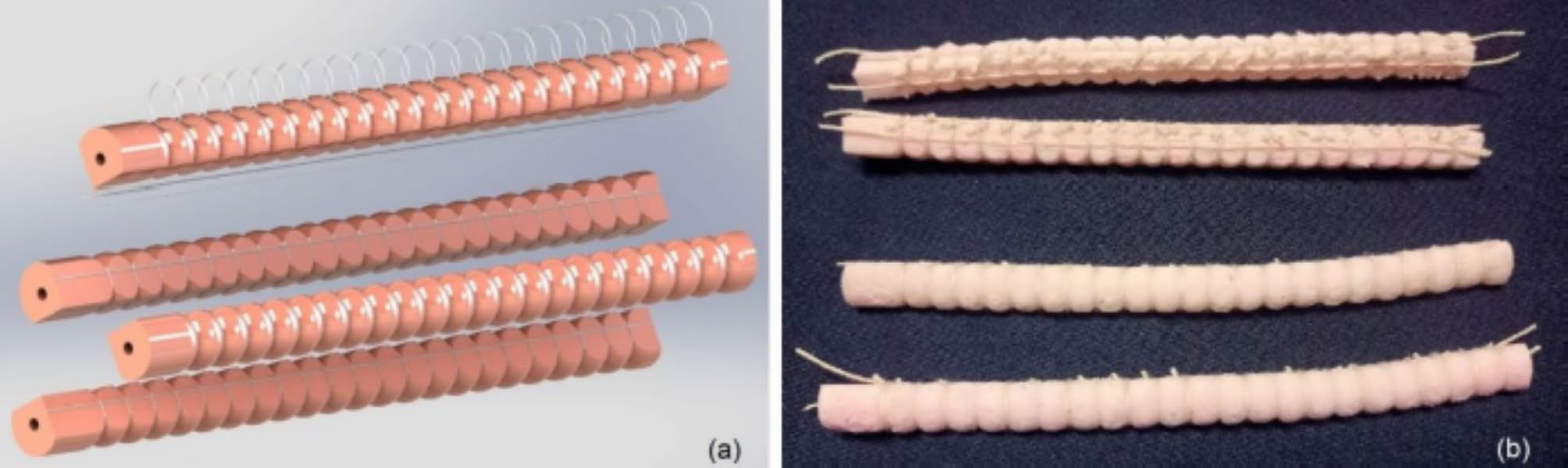
(**a**) 3D modeled reinforced actuator version. (**b**) Introducing fibers during fabrication.

**Fig. 12:**
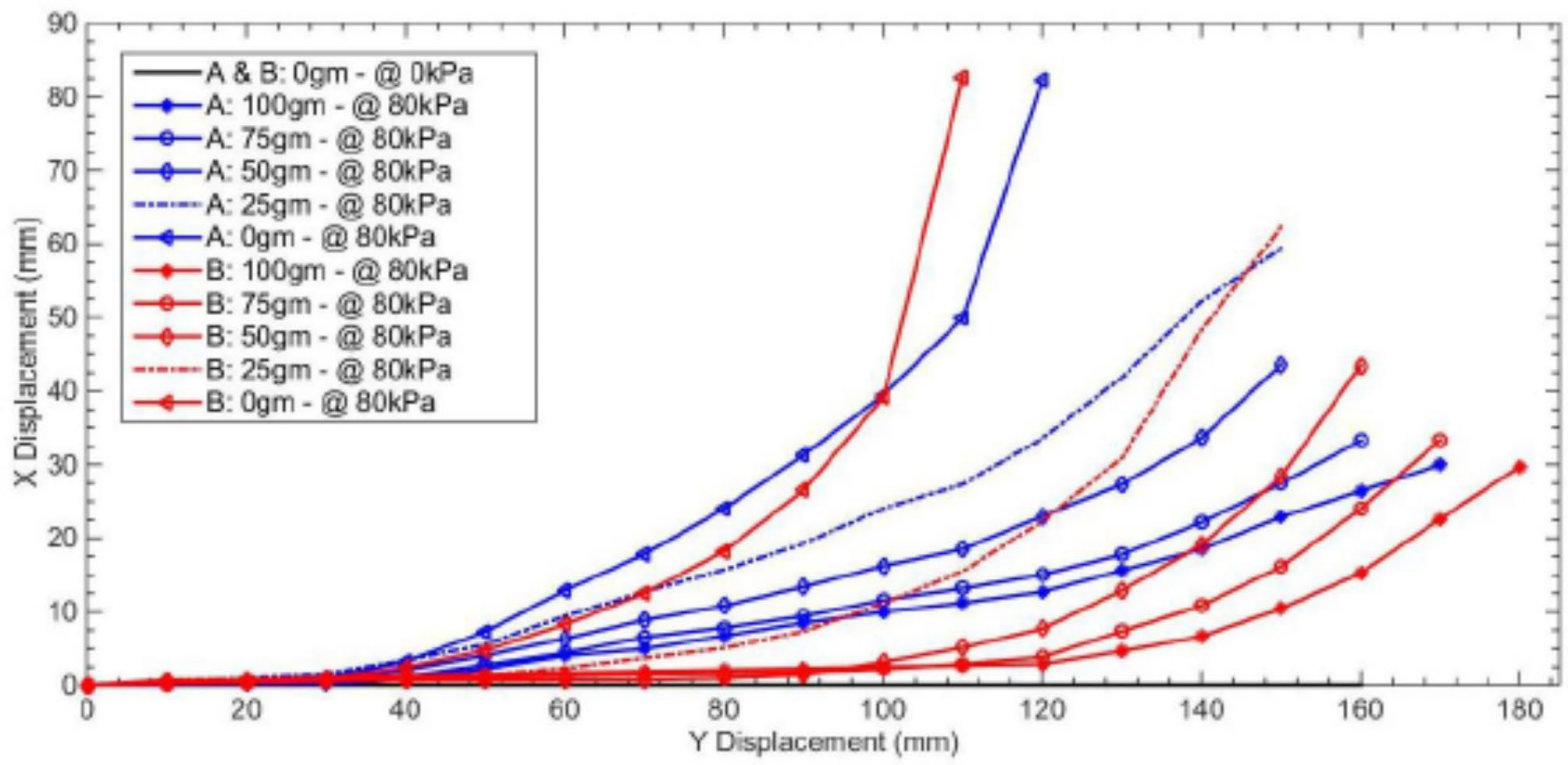
Actuators load testing for the reinforced actuator represented by the letter A and the plain actuator represented by the letter B.

During testing the actuator with wall thickness of 2.5 mm, the maximum reliable pressure was degraded to 80 kPa; however, observation when increasing the pressure more starting from 85 to 110 kPa is attained with sudden failure. Accordingly, the simulation design values are degraded after experimental implementation; that, the maximum operating pressure is degraded from 100 to 80 kPa, with a margin of safety of at least 5 kPa before actuator’s failure. In order to limit the observed unwanted longitudinal strain and deformations, found during experimental testing, a reinforced actuator version is developed as illustrated by Fig. 11. The reinforcement is performed using cotton fibers of approximate diameter of 0.5 mm. Fibers are embedded in longitudinal arrangement to the interface faces of attached modules forming the actuator. Additionally, radially arranged fibers are used to limit unwanted hoop strain at the chamber’s overlap sections along every module’s network. The two actuators’ versions are tested by attaching different loads of 25, 50, 75 and 100 g to the actuator end and applying 80 kPa to dual adjacent actuators. Curves illustrated in Fig. 12 show the actuators’ bending motion as X–Y displacement; where the reinforced actuator is represented by the letter A in blue color and the plain actuator is represented by the letter B in red color. The higher Y displacement in the curves reflects higher longitudinal displacement; this appears clearly with increasing the weight as the Y-axis is the vertical axis along the gravity direction. Additionally, the more rounded curvature accompanied by low X displacement reflects higher radial elongation; since this behavior is due to exceeded chambers’ volume strain along the actuated network. By comparing the red curves to the blue ones, the actuator behavior for the plain actuator shows more longitudinal elongation with increasing the load, in addition to exceeded rounded shape curves which reflects the unwanted strain; for more clarification, at lifting 100 g weight and at same X displacement, the Y displacement of the plain actuator increased by around 5% in comparison to the reinforced actuator.

**Fig. 13:**
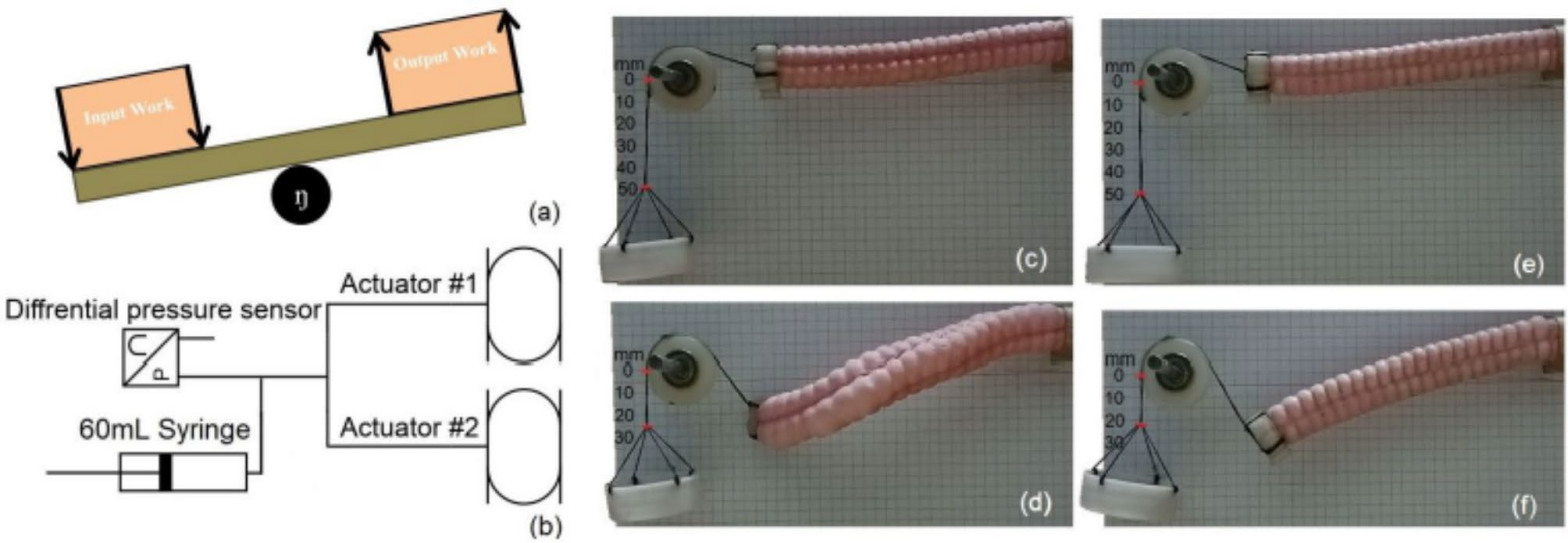
(**a**) Conceptual schematic for the energy balance. (**b**) Schematic diagram for energy test comparison. (**c**) Plain actuator in start position. (**d**) Plain actuator tested to lift 25 g by 25 mm with pressurizing two adjacent networks by 78 kPa using volume of 60 ml. (**e**) Reinforced actuator in start position. (**f**) Reinforced actuator tested to lift 25 g by 27.5 mm with pressurizing two adjacent networks by 78 kPa using volume of 45 ml.

The unwanted strain reflects extra energy consumption, investigating more the performance and efficiency of the plain actuator to the reinforced actuators; a test is performed in which a 25 g weight is fixed to the end of each actuator. The schematic in Fig. 13a shows a conceptual clarification for the energy balance where the work due to pressurized air is the input work to the actuator and the lifting is the output work. Figure 13b clarifies the test arrangement; where a 60 mL syringe is used to determine the used air volume in the test and a pressure sensor will measure the pressure, accordingly the input energy can be recorded. The actuator is fixed on a stand in horizontal position. A pulley is used to guide a wire lifting a vertically hanged 25 g weight; the other end of the wire is fixed to the actuator’s end, which is kept in horizontal position. Considering the change of the elevation of the weight multiplied by the 25 g weight is corresponding to the output energy, which is the work done to lift the 25 g weight, Fig. 13c, d show the plain actuator tested to lift 25 g by 25 mm with pressurizing two adjacent networks by 78 kPa using volume of 60 ml. However, Fig. 13e, f show the reinforced actuator tested to lift 25 g by 27.5 mm with pressurizing two adjacent networks by 78 kPa using volume of 45 ml. First observation was the apparent unwanted deformation in Fig. 13d which is difficult to be controlled. Additionally, by calculating the efficiency for both actuators’ performances.

$$\eta=\frac{\text{o}\text{u}\text{t}\text{p}\text{u}\text{t}\:\text{w}\text{o}\text{r}\text{k}}{\text{i}\text{n}\text{p}\text{u}\text{t}\:\text{w}\text{o}\text{r}\text{k}}=\frac{\text{M}\:\left(\text{W}\text{e}\text{i}\text{g}\text{h}\text{t}\right)\times\:\text{d}\:\left(\text{D}\text{i}\text{s}\text{p}\text{l}\text{a}\text{c}\text{e}\text{m}\text{e}\text{n}\text{t}\right)}{\text{P}\:\left(\text{P}\text{r}\text{e}\text{s}\text{s}\text{u}\text{r}\text{e}\right)\times\:\text{V}\:\left(\text{V}\text{o}\text{l}\text{u}\text{m}\text{e}\right)}$$

**Fig. 14:**
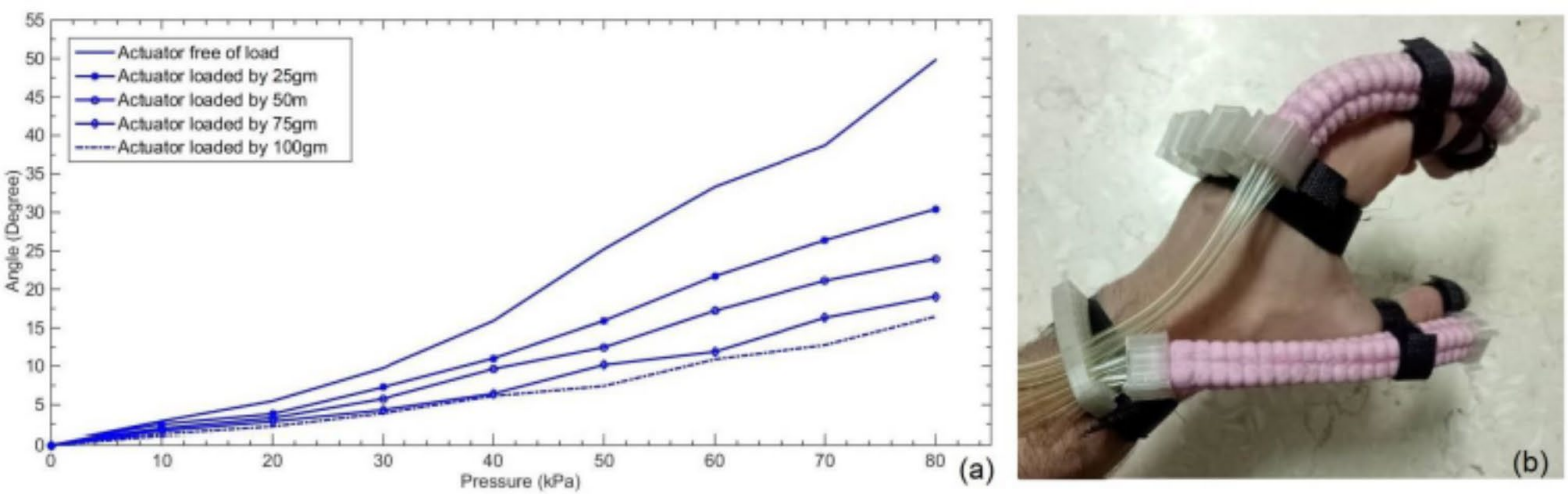
(**a**) Reinforced actuator’s pressure to bending angle results when different loads are lifted by the actuator’s end. (**b**) Employing the actuator in hand wearable glove.

The reinforced actuator shows higher efficiency over the plain actuator which reaches (46%). Correlating the reinforced actuator version performance, Fig. 14a shows bending angles response to the applied pressure is illustrated with different loads being attached to the actuator’s end, used loads are no-load, 25, 50, 75 and 100 g, the aim of such correlation is to show the range of grasping that the actuator can achieve; where, the maximum records of the parameters characterize the actuator are at the 80 kPa; so, the actuator can bend up to angle of 16 degree while lifting load of 100 gm exerting 0.13 Nm torque. Finally, Fig. 14b illustrates the actuator employed in a primary hand wearable glove arrangement. At the end Table 1 shows parameters comparison for some literature work in comparison to the proposed reinforced modular actuator.

**Table 1:** Actuators characteristics comparison.

References	Design	Motion	Max pressure (kPa)	Max force (*N*) or torque (Nm)
^1^	Soft hoop-reinforced pneumatic actuator	Flexion	200	2.5 to 3 N
^3,11^	Low-Cost Fabric-Based Flat Pneumatic Actuators	bi-directional flexion and extension	70	0.31 Nm
^12^	Fiber-reinforced soft bending pneumatic artificial muscles	counteracting tremor	655	2 N
^13^	Fiber reinforcement elastomeric chambers soft actuator	Flexion	400	8 N
^14^	Double segmented soft-elastic composite actuator with torque compensation layer	Flexion and extension	250	N/A
^15^	Novel monolithic fabrication method soft actuator	Flexion	526	N/A
^20^	Bellows structured pneumatic soft actuator single chamber and dual chamber	Single bending	80	N/A
Current work	Fiber reinforced Pneu-Net modular soft actuator	Flexion, extension, adduction and abduction	80	0.13 Nm

## Conclusion and future work

In this paper, a modular design based soft Pneu-Net actuator is developed inspired by elephant trunk. The actuator is developed to be employed in rehabilitation hand glove providing the four finger’s motions which are the flexion, extension, adduction and abduction. The new inputs that the actuator design introduces can be summarized in its modular design which will facilitate the reproduction of the actuator in a tailored manner for every case, and introducing a single compound actuator that can provide four DoF of bending motions. In addition to that, a simplified non-standard biaxial tension test is proposed facilitating the conduction of hyper-elastic material test table that can be used in FEM software material identification. The simulation result’s accuracy can be accepted to the level of simplification, which facilitates the reproduction of the actuator with the available material even if its specification is not defined. The selection of an optimum actuator’s wall thickness is depending on the value of pressure that the actuator will withstand which impact the forces that the actuator can produce; this can be tested by the simulation. A reinforced actuator version is introduced which utilize cotton fibers to limit the unwanted longitudinal extension of the actuator and the radial inflation of the chambers, which resulted in better performance of the actuator. The enhanced performance is shown in the bending locomotion and the energy efficiency that the reinforced actuator shows higher efficiency over the plain actuator reaches (46%).

The work to be continued in the future can be dedicated to three main points; the first point is the non-standard simplified material test, since the results should be enhanced more to be more accurate. The second point is enhancing the actuator’s energy efficiency and improving the test methodology to be more indicative and eliminate loses resulted from the power test however the used test was sufficient to compare two different actuators and define the more efficient one. Finally, the employed application for the actuator in the rehabilitation hand glove and getting more testing results in this regards to validate the actuator with subjects.

## Data Availability

The datasets used and/or analysed during the current study available from the corresponding author on reasonable request.
